# Workforce participation, health and wealth inequality among older Australians between 2001 and 2015

**DOI:** 10.1186/s13690-022-00852-z

**Published:** 2022-03-31

**Authors:** Huong Dinh, Lyndall Strazdins, Tinh Doan, Thuy Do, Amelia Yazidjoglou, Cathy Banwell

**Affiliations:** 1 Australian Treasury, Canberra, Australia; 2 Research School of Population Health, the Australian National University, Canberra, Australia

**Keywords:** Older people, Employment, Health, Economic inequality, Australia

## Abstract

**Background:**

Australians born in 2012 can expect to live about 33 years longer than those born 100 years earlier. However, only seven of these additional years are spent in the workforce. Longer life expectancy has driven policies to extend working life and increase retirement age; the current Australian policy, which has increased the eligibility for the pension from 65 to 67 by 2023, assumes that an improvement in longevity corresponds with an improvement in healthy life expectancy. However, there is mixed evidence of health trends in Australia over the past two decades. Although some health outcomes are improving among older age groups, many are either stable or deteriorating. This raises a question of how health trends intersect with policy for older Australians aged from 50 to 70. This paper considers the interplay between older workers’ health and workforce participation rates over the past 15 years when extended workforce participation has been actively encouraged.

**Methods:**

We compared health and economic outcomes of the older people in following years with the base year (start of the study period), adjusting for some key socio-economic characteristics such as age, sex, ethnicity, education and equivalized household income by applying the Random effects estimator with maximum likelihood estimation technique.

**Results:**

We find that regardless of increasing longevity, the health of older adults aged between 50 and 70 has slightly deteriorated. In addition, health gaps between those who were working into their older age and those who were not have widened over the 15-year period. Finally, we find that widening health gaps linked to workforce participation are also accompanied by rising economic inequality in incomes, financial assets and superannuation. With the exception of a small group of healthy and very wealthy retirees, the majority of the older Australians who were not working had low incomes, assets, superannuation, and poor health.

**Conclusions:**

The widening economic and health gap within older population over time indicates a clear and urgent need to add policy actions on income and health, to those that seek to increase workforce participation among older adults.

**Supplementary Information:**

The online version contains supplementary material available at 10.1186/s13690-022-00852-z.

## Background

In Australia, life expectancy at birth for people born in 2012 compared to those born at Federation (1901) has increased approximately 33 years. Recent trends indicate that only seven of these additional 33 years are spent in the workforce [1]. The discrepancy between increased life expectancy and length of labour force participation, in combination with an aging population, has ramifications for government spending and the financial security of older Australians.

The current Australian “Age Pension” policy [2], which has increased the eligibility for the pension from 65 to 67 by 2023, assumes that an improvement in longevity corresponds with an improvement in healthy life expectancy. However, there is mixed evidence from two decades of health trends in Australia, which indicate that while some health outcomes are improving among older age groups, many are either stable or deteriorating. There has been a 2–3% improvement in arthritis for older age groups (45–54, 55–64 and 65–74) in 2014–15 when compared with the same age-group in 2004–05 [3, 4]. Cancer seems to remain stable but rates of diabetes, heart, stroke and vascular disease are increasing [3, 4]. This mixed evidence raises the question of how health trends intersect with policy for older Australians aged from 50 to 70.

This paper considers the interplay between older workers’ health and workforce participation rates between 2000 and 2015, during a period when older workers were actively encouraged to keep working. The study aimed to describe the health profile of this population over time using the Household Income and Labour Dynamics in Australia survey (HILDA) data, and to examine what drives the widening health gap between those remaining in the workforce as they age, and those who do not. This will provide new insights into the interrelationships between health, employment and economic resources among older workers who are the target of government efforts to extend their working life and reduce their costs on the economy. The study provides further insight into potential worsening of economic and health inequality amongst older people, because for many living longer does not necessarily mean living with better health. Our findings contradict the assumption all older Australians are achieving longer healthier lives and are therefore able to extend their working lives, which was used to support the current Australian retirement age extension.

### Healthy life expectancy and longevity

An ageing population may impact on national employment, productivity, public finance, health care requirements and service delivery all of which in turn, affect population health, growth and social equity [5]. Consequently, it is important to understand the dynamics of population ageing. Internationally, the health of older adults, or their healthy life expectancy, has not generally improved at the same rate as increasing life expectancy at age 65 although the picture varies by country [6]. For example, in the Netherlands a healthy life, defined as without major illness or disability or a morbidity-free life expectancy as a percentage of total life expectancy or longevity, decreased significantly from 69.2% in 1989 to 63.3% in 2000 for women, (from ages 55.3 to 51.0 years), and from 74.2 to 71.4% for men (from ages 54.7 to 53.9 years) [7]. Similarly, in Australia, the proportion of adults living a healthy life remained constant between 2003 and 2011 at around 88% despite longevity increasing [8]. The discrepancy between longevity and good health has significant consequences for older workers.

The health of older workers is a key driver of their ability to continue working, with the prevalence of chronic conditions rising as people age [9]. For example, approximately 42% of workers in Britain aged 50–64 live with a health condition or disability. In Australia, six out of ten workers reported involuntary retirement due to poor health [10, 11]. Due to the significant impact of health on employment, there are major health disparities between those who work or can work and those who cannot. For example, 27% of Australians without chronic disease are more likely to work beyond the recommended retirement age compared to 23% people with a chronic disease [12]. A diagnosis of a common chronic disease such as diabetes, for example, is associated with a 30% increase in the rate of labour-force exit in Europe and the US [13]. As working-class employees age, they face a higher rate of poor health and disability, due to occupational hazards and physically demanding jobs [14]. Furthermore, job loss or unemployment is associated with an increased risk in mortality and cardiac events [15]. Older workers who remain employed for longer are healthier, with less physical incapacity or disease than their non-employed or involuntarily retired counterparts [12, 16]. The widening health outcomes between those who are able to work and those who are not in then older population, is accompanied by a growing inequality in their economic resources [13, 17].

Gains in life and healthy life expectancy are disproportionately occurring among more affluent individuals [17]. Health and income form part of a virtuous cycle over the life-course which, in older age, mean that good health enables a life time benefit of longer work force participation and accumulated income and wealth, whilst the healthy life expectancy of lower-income workers remained stable or in some cases has declined [17]. In Australia, those in the highest socioeconomic group expect seven more years of full health for men and 4.8 more years for women than those in the lowest socioeconomic group [8]. Thus there is an increasing number of older, healthier and highly educated workers remaining employed and able to earn income for longer whilst those with poorer health, less education and less earning capacity have less ability to prolong their workforce participation [14]. Despite policy to encourage the participation of older adults in the work force, health becomes an increasingly important barrier for extending working lives and therefore an influential factor for the expansion of socio-economic inequalities in the older population.

Policy targets that treat all older population the same are therefore likely to be ineffective, due to rising health and economic inequality amongst older people [14, 17]. For example, there are some people who are wealthy and healthy, who may not want to work or choose to retire early, while there are others who need income and employment but their poor health restricts them from labour market participation. A monolithic policy for older populations could have negative unintended consequences on some groups. This study decomposes the population of 50–70 year olds into subgroups based on their economic resources and health to help uncover detailed interrelationships between health and employment among this population. Understanding how the older population fares in terms of their health and wealth is crucial for informing policy relating to healthy and productive aging.

We hypothesise that:

- H1: Although there has been an increase in labour-force participation of older workers the health profile of employed older workers has not improved. That is, improved workforce participation rate does not necessarily reflect only health improvements, but also other reasons e.g. financial needs.
- H2: Health gaps between the employed and non-employed older population have also widened.
- H3: The widening health gap is accompanied by widening income, superannuation, and net financial asset gaps.

## Materials and methods

### Data and sample

#### Data

To test the hypotheses above, we used the first 15 waves of the Household Income and Labour Dynamics in Australia (HILDA) data set which is highly valued for informing policy relating to healthy and productive ageing [18]. This survey, which commenced in 2001, is a nationally representative household-based panel study of Australian adults aged 15 years and over. Each wave consists of about seven thousand households or more and includes at least 17,000 household members. Those living outside Australia, such as diplomats, residents of institutions and people living in remote areas were excluded. The survey asked respondents about their employment, economic resources, family circumstances, health and socio-economic characteristics through face-to-face interviews and self-complete questionnaires. Response rates were consistently high, with a retention rate of 86% in wave 2, and above 90% in following waves [19].

#### Sample

Our current study focuses on HILDA survey respondents aged 50 to 70 in each wave who reported on physical functioning, mental health, general health, health conditions, and other individual characteristics and socio-economic factors. We focussed on this age group because they are transitioning into retirement at a time when health and employment interplay most during life course. Because people age over time, this sample automatically includes new people who entered the age bracket (50–70) and excludes those who exited the age upper bound in later years. The final sample includes 60,723 observations of which 3039 unique (*N* = 28,787) are males and 3138 unique (*N* = 31,936) are females over 15 years. Because of the age cut-offs, attrition and recruitment of new respondents, the number of observations varies across waves.

### Measures of health outcomes

In this paper, we focused on four key self-reported health outcomes: mental health, physical functioning, general health, and number of health conditions.

*Mental health* was constructed from five items of the Short Form 36 (SF-36) health status questions [20]. The mental health scale is widely used in population-based surveys, performing best among the eight SF-36 health scales in cross-sectional and longitudinal analyses of patients with clinical distress [21]. *Physical functioning* was constructed from 10 items of the SF-36, which assess physical capacities in various activities. General health was constructed from five items which assessed self-assessed general health. These health measures were re-coded on a 0-to-100 scale with a higher score meaning better health (see, [19]) (Table 1).

**Table 1:** Health measures and definition

Health measures	SF-36 subcomponents and scale
Mental Health (score 0–100)	Been a nervous person? (1–6; 1: all of the time − 6 none of the time)
	Felt so down in the dumps nothing could cheer you up (1–6)?
	Felt downhearted and blue (1–6)?
	Felt calm and peaceful (1–6)?
	Been a happy person (1–6)?
Physical functioning (score 0–100)	Vigorous activities (1–3: 1 limited a lot, 2 limited a little, 3 not limited at all)
	Moderate activities (1–3)
	Lifting or carrying groceries (1–3)
	Climbing several flights of stairs (1–3)
	Climbing one flight of stairs (1–3)
	Bending kneeling or stooping (1–3)
	Walking more than one kilometer (1–3)
	Walking half a kilometer (1–3)
	Walking 100 m (1–3)
	Bathing or dressing yourself (1–3)
General Health (score 0–100)	Self-assessed health (from 1-excellent to 5-poor)
	Get sick a little easier than other people (1-definitely true to 5-definitely false)
	As healthy as anybody I know (1–5)
	Expect my health to get worse (1–5)
	My health is excellent (1–5)

Apart from the three main health measures above, we also examined the ‘*number of health conditions’* which was collected from wave three (2003) onwards, respondents were asked if they had any specific long-term health conditions in the list of 17 health states (yes/no questions). For the details of these variable and its components, see [19]. A total health condition index was created by taking the sum of all (17) incurred health conditions.

### Economic outcome variables

In this paper, we also considered some key economic outcomes that may be associated with the older population’s employment and health. They are: (1) *regular private income,* the sum of a financial year regular market income (wages and salary, business income, investment income, and regular private pension income), and regular private transfers; (2) *income from all sources,* the sum of regular private income and Australian and foreign pensions, and social welfare benefits (but irregular income such as one-off payment, lottery prize… is excluded); (3) *superannuation,* the sum of retiree and non-retiree superannuation, and it is accumulated over time and linked to employment. Superannuation data is available in only waves 2, 6, 10 and 14 only; (4) *net financial assets* exclude property assets and property debts, and business assets and business debts. We excluded property and business assets as they are not directly related to wage labour market outcomes. Net financial assets are those remaining after debts e.g. credit card debts, overdraft account, overdue bills, capture superannuation, saving on bank accounts, bank account balance, cash and equity investments, trust funds and life insurance.

### Covariates

In the prediction models for health outcomes, income, net financial assets, and superannuation, we adjusted for gender, ethnicity, age, education, and equivalized household income to remove the influence of these factors on the outcomes because they were not attributable to working status. All variables in monetary terms were discounted to 2002 price level using the annual Consumer Price Index (CPI). Below are definitions of variables and covariates.

*Gender* takes value 1 for male and 0 for otherwise. *Ethnicity* takes four values 1 for non-Indigenous Australian, 2 for Aboriginal and Torres Strait Islander Australian, 3 for other English-speaking migrants, and 4 for non-English speaking migrants. *Age* is a continuous variable ranging from 50 to 70 as we focused on the older labour force. *Education* is a dummy variable taking value 1 for tertiary education, and 0 for otherwise. *Equivalized income* per capita*,* computed from household financial year disposable regular income, is the household’s financial year gross regular income after income taxes. As this variable was measured at household level, we adjusted for the household composition using the modified OECD equivalence scale^1^.

### Statistical approach

Individual and socio-economic characteristics were used as predictors for health outcomes in the models, as follows:

1 $${y}_{it}={\mathbf{X}}_{it}\boldsymbol{\upbeta} +{\boldsymbol{\upvarepsilon}}_{it}\kern4em where\kern1.5em {\boldsymbol{\upvarepsilon}}_{it}={\alpha}_i+{\eta}_{it}$$

*y*_it_ is health outcomes for individual *i* in year *t*, *X* is a vector of individual and household characteristics such as age, gender, ethnicity, education, and equivalized income per capita in logarithm. We also controlled for wave or time effect in the model. Instead of using raw health outcomes, we adjusted for these important factors affecting individual’s health outcomes, however, our model does not aim to examine a causal relationship between the covariates and health outcomes.

The error terms (ε_it_) has two parts, a time-invariant component (*α*_*i*_*)* that differs across individuals and a component (*η*_*it*_*)* that is independent of both time and individuals. The individual component *α*_*i*_ may be correlated with the variables in **X**_*it*_ (fixed effects model, FE) or uncorrelated with **X**_*it*_ (random effects, RE).

We employed the RE estimator with maximum likelihood estimation technique. Two-level maximum likelihood regression was used: the first level assumes fixed effects for demographic covariates while the second assumes random effects within individuals. This approach allows inclusion of both within- and between-individual effects and addresses limitations present in the fixed-effect models, which only examines within-individual effects [22, 23]. Our approach overcomes problems of imprecise estimates with large standard errors if predictors vary between individuals but doesn’t vary over time, e.g., sex, ethnicity, or does not vary or vary very little over time e.g. education [24]. The prediction of the economic outcomes such as income, net financial assets and superannuation used the similar model for health outcomes as in Eq. (1), but we did not control for the equivalized household income as we did in the health models.

The analyses were stratified by working status (being employed versus being non-employed) to predict health outcomes for each group in order to examine how the predicted health outcomes by each group changed over the considered period (2001–2015). The model for each health outcome was conducted for the population aged 50–70. The relative changes in health outcomes were captured by the time-specific coefficients. The year 2001 was set as a baseline for comparison with the following years’ health outcomes. For number of health conditions, the base year was 2003, as this health variable was not collected before 2003. For example, if a health outcome in 2015 was better (or worse) than in the baseline of 2001, then the coefficient of year dummy of 2015 was positive (or negative). The estimated changes of health and economic outcomes over time were illustrated in the graphs in coming sections.

## Results

### Which older Australians are employed?

Table 2 describes the socio-economic characteristics of the older population by their working status in 2001 and 2015. On average, employed older Australians were about five years younger than their non-employed counterparts. The age gap is quite stable across 2001 and 2015, although the average age incrementally increased over time for both the employed and non-employed older population. There were more males in the older employed group in both years.

**Table 2:** Descriptive summary, older Australian population aged 50–70, 2001 vs. 2015

	2001			2015		
	Non-employed	Employed	T-test for difference	Non-employed	Employed	T-test for difference
	Mean [CI]	Mean [CI]	(*P*-value)	Mean [CI]	Mean [CI]	(*P*-value)
Age	61.2 [60.9–61.5]	56.0 [55.8–56.2]	0.0000	62.3 [62.1–62.6]	57.01 [59.0–59.3]	0.0000
Gender (Male =1) (% of male)	41.1 [38.8–43.4]	56.4 [54.1–58.7]	0.0000	38.9 [36.8–41.0]	52.0 [50.2–53.7]	0.0000
Tertiary education (%)	33.9 [31.7–36.1]	51.2 [49.1–53.7]	0.0000	49.5 [47.4–51.7]	69.6 [68.0–71.2]	0.0000
*Ethnicity (%)*						
[1] Australian born	62.2 [59.6–64.4]	70.6 [68.4–72.7]	0.0000	68.0 [66–70]	72.8 [71.2–74.3]	0.0002
[2] Aboriginal	1.38 [0.84–1.92]	0.79 [0.38–1.20]	0.0870	2.1 [1.5–2.7]	1.4 [0.96–1.77]	0.0324
[3] English speaking migrants	15.3 [13.7–17.0]	14.8 [13.2–16.5]	0.6455	12.9 [11.5–14.4]	12.8 [11.7–14.0]	0.9323
[4] Non-English speaking	21.0 [19.1–22.9]	13.8 [12.2–15.4]	0.0000	16.8 [15.2–18.4]	13.0 [11.8–14.1]	0.0001
Mental health (scaled 0–100)	72.2 [71.3–73.1]	78.1 [77.4–78.9]	0.0000	72.0 [71.2–72.9]	77.2 [76.6–77.8]	0.0000
Physical functioning (0–100)	66.8 [65.4–68.1]	83.7 [82.8–84.6]	0.0000	69.0 [67.8–70.2]	84.7 [84.0–85.3]	0.0000
General health (scaled 0–100)	58.8 [57.6–60.1]	72.1 [71.2–73.0]	0.0000	57.2 [56.1–58.3]	68.7 [68.0–69.4]	0.0000
No of health condition (0–15)^a^	0.76 [0.72–0.79]	0.27 [0.25–0.30]	0.0000	0.86 [0.82–0.89]	0.31 [0.29–0.33]	0.0000
*Self-assessed health (%)*						
[1] Excellent	7.47 [6.2–8.7]	13.47 [11.8–15.1]	0.0000	4.48 [3.57–5.40]	8.2 [7.2–9.2]	0.0000
[2] Very good	22.5 [20.5–24.5]	40.7 [38.3–43.0]	0.0000	22.2 [20.3–24.0]	35.6 [33.8–37.3]	0.0000
[3] Good	33.99 [31.7–36.2]	34.6 [32.3–36.9]	0.6983	37.6 [35.5–39.8]	41.1 [39.3–42.9]	0.0158
[4] Fair	26.3 [24.2–28.4]	9.6 [8.2–11.0]	0.0000	26.1 [24.2–28.1]	14.1 [12.8–15.4]	0.0000
[5] Poor	9.7 [8.3–11.1]	1.6 [1.0–2.2]	0.0000	9.6 [8.3–10.9]	1.0 [0.64–1.36]	0.0000
Regular private income	8.8 [7.9–9.6]	44.6 [42.3–46.8]	0.0000	13.7 [12.4–14.9]	54.5 [52.6–56.3]	0.0000
Income from all sources	14.9 [14.2–15.7]	45.4 [43.1–47.6]	0.0000	21.4 [20.1–22.6]	55.5 [53.7–57.4]	0.0000
Household net worth	504.8 [467–542]	812.2 [752–872]	0.0000	754.6 [709–800]	925.3 [888–963]	0.0000
Net financial assets ^b^	203.8 [183–224]	319.5 [259–344]	0.0000	334 [302–365]	395 [373–416]	0.0012
Superannuation^b^	100.2 [89.6–111]	180.2 [168–192]	0.0000	213.6 [195–232]	272 [259–285]	0.0000

All monetary variables were discounted to 2002 price. The Economic outcome variables were in AU$1000

^a^Data available from wave 3 onwards

^b^Data were available in every four years since 2002

Employed older adults were more educated than their non-employed counterparts, particularly in 2015. They were more likely to be Australian born, and less likely to be Aboriginal or non-English migrants. Although there were some changes in relative ethnic composition over time, the overall ethnic patterns did not change.

Table 2 also presents unadjusted health and economic outcomes by the working status. Older employed adults were healthier than those not in employment in all health measures such as mental health, physical functioning, general health, number of health conditions, and self-assessed health measure. The health gap between the two groups persisted over the period. Older workers earned more, and had significantly higher superannuation and net financial assets than those who were not in the labour force. In 2001, on average, regular private income was almost 5 times higher among those who were employed as those who were not, and about 3 times higher if government supports such as pension and social welfare benefits are included. The employed group also had about 88% more in superannuation and 57% more in net financial assets than the non-employed group in 2001, the gaps narrowed in 2015 but remained large.

In summary, those in the age cohort (50 to 70 years) who were still working were more likely to be male, relatively younger on average, healthier, more educated, with higher incomes, net financial assets, and superannuation than those who were not employed in the same age cohort.

### Labour market participation rate (%) among older Australians 2001 to 2015

**Fig. 1:**
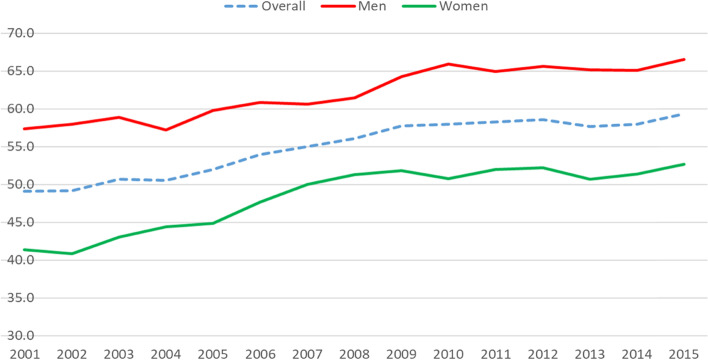
Labour market participation rate over time by gender, aged 50–70. Source: Authors’ estimation from HILDA 2001–2015

The workforce participation rate of older Australians aged 50–70 also increased steadily over the past 15 years commencing 2001 (Fig. 1), from 49% in 2001 to 59% in 2015. Participation increased for both men and women. Although the workforce participation rate is considerably higher for men, the gender participation gap between men and women slightly decreased, from 17% in 2001 to 14% in 2015. This rising participation rate implies that the policy aiming at encouraging older people to participate or to stay in the labour market longer has been a success.

### Little evidence of improvement in health of the older population

The rise in the older workers’ labour force participation signals success for policies aiming to increase labour supply. However, this raises the question of whether or not rising participation reflects improvement in health. Given the mixed evidence to date we hypothesise that health profile of employed older workers may not have improved [H1]. In this subsection, we look into changes in health of the older population over the period to answer such question.

**Fig. 2:**
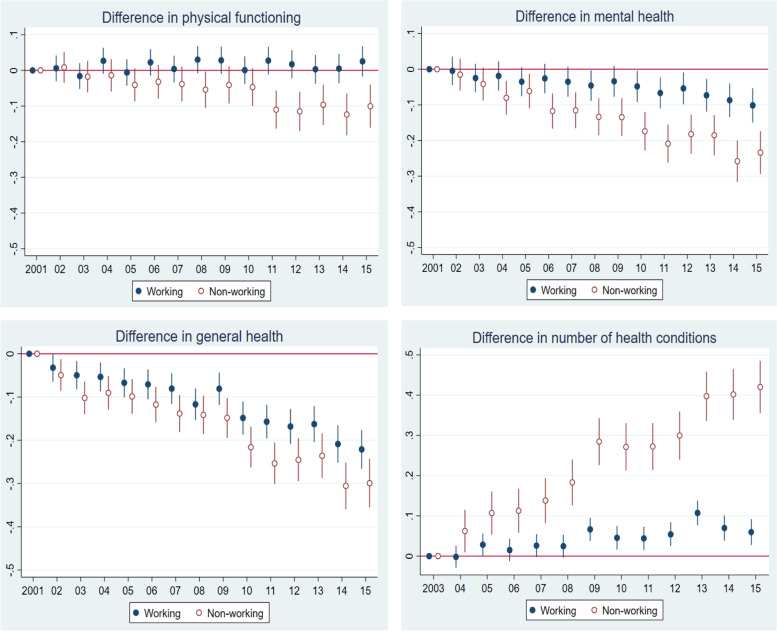
Relative change of health outcomes from 2001 (base year) to 2015, Australians aged 50 to 70. Notes: 2001 is the start year that the health data are available, otherwise indicated. All the health scores/measures were standardised by its mean and standard deviation in the 50–70 sample in order to put health outcomes in the same range figures for convenient comparison. All health outcomes were adjusted for age, gender, ethnicity, education and income in the maximum likelihood RE model

In Fig. 2, we compared health outcomes for the following years with the base year 2001, adjusting for some key socio-economic characteristics such as age, sex, ethnicity, education and equivalized household income (in the random effect model-eq. 1). It shows that for the older population, there is a gradual decline in all health outcomes over the study period, except that the physical functioning of employed older adults has remained stable.

The RE maximum likelihood estimates using Eq. (1) shows that compared to the baseline in 2001, the mental health of similar employed people (in terms of age, sex, ethnicity, education and equivalized household income) worsened by 0.1 standard deviations (*p* < 0.01) by 2015. Their general health declined by 0.22 standard deviations (*p* < 0.01), and the number of health conditions increased slightly by 0.06 standard deviations (*p* < 0.01)^2^. Only their physical functioning improved (but was statistically insignificant) from baseline health. For non-employed people, their health outcomes deteriorated faster than their employed counterpart’s over the same period. Non-employed people’s mental health was 0.23 standard deviations lower (*p* < 0.01), physical functioning 0.1 standard deviations lower (*p* < 0.01), general health 0.3 standard deviations lower (*p* < 0.01), and number of health conditions 0.42 standard deviations higher (*p* < 0.01).

Overall, regardless of working status, in most instances we observed health deterioration. However, employed people are clearly healthier than non-employed people, and their health outcomes have deteriorated more slowly than those who were not employed over the same period.

### Widening health gaps

**Fig. 3:**
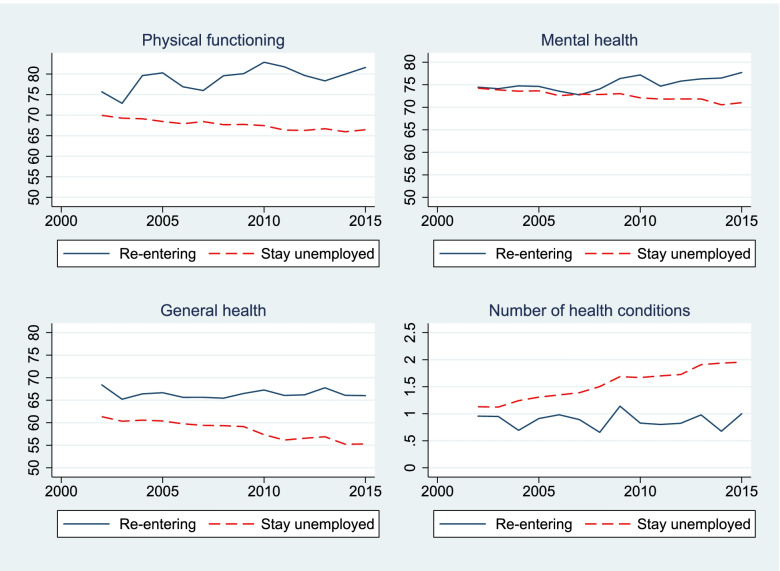
Health of the re-entering workers vs. the ‘stay unemployed’ Australian population, aged 50–70, 2001–2015. Note: Estimates were adjusted for age, sex, ethnicity, education, equivalized income per capita using the random effect maximum likelihood estimator

**Fig. 4:**
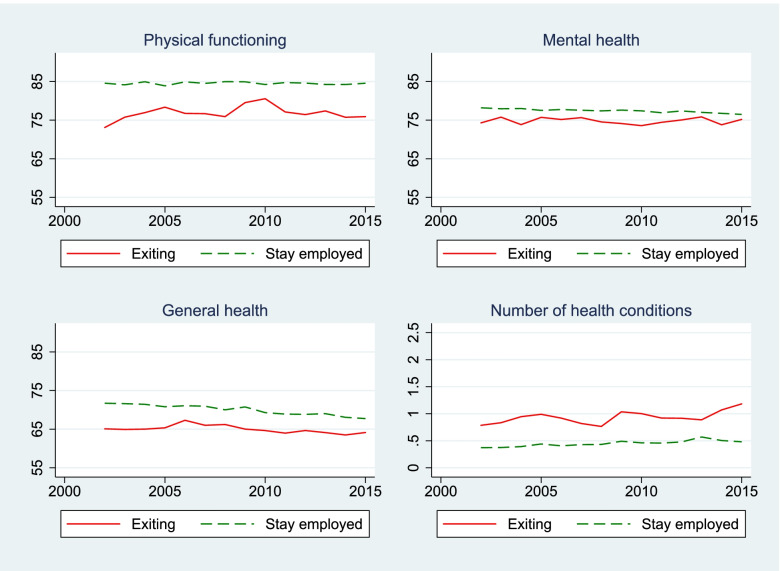
Health of the exiting workers vs. to the ‘stay employed’ Australian population, aged 50–70, 2001–2015. Note: Estimates were adjusted for age, sex, ethnicity, education, and equivalized income per capita using the random effect maximum likelihood estimator

In this subsection, we considered the health changes over time among older employed and non-employed people, and propose contributors to the widening gap. Though employed older adults’ health did not improve over time, the health of the non-employed people deteriorated faster. This has generated a widening health gap between the two groups, as proposed in Hypothesis 2 (Fig. 2). This is also confirmed by a statistical test which shows that the interaction terms between the working status (1/0) and the time variable in the health prediction Eq. (1) are statistically significant (the test results are available upon request). The health selection process may operate i.e. better health supports the non-employed returning to the labour market (Fig. 3), while poorer health is found amongst employed people exited the workforce (Fig. 4).

We observed widening health gaps between those who were working and those who were not, begging the question - what contributes to such a widening gap? To answer this question, we broke down the sample into four groups. These were: re-entering the workforce, remaining employed, exiting the workforce, and remaining non-employed. People who are working in a specific year either remained employed or became non-employed in the following year. For example, among 1733 people aged 50–70 who were employed in 2001, 1538 people stayed employed and 195 people left employment (exited the workforce) in 2002. For people who were not employed in a specific year, we tracked their employment status in the following year. They were either not employed or returned to the workforce in the following year. For example, among 1566 people aged 50–70 who were not employed in 2001, 1471 people remained non-employed and 95 people became employed in 2002.

In Fig. 3, we compared health outcomes by employment transition status. Figure 3 (and also Fig. 4) was adjusted for age, sex, ethnicity, education, equivalized income per capita, and time effect using the random effect maximum likelihood estimator. It clearly shows that the “re-entering” group has better health outcomes than the “stay unemployed” (Fig. 3). The health scores for physical functioning, mental health and general health of the re-entering people were considerably higher than the “stay unemployed” people, and they also have a significantly lower number of health conditions. Moreover, the health gap between these two groups has widened. Departure of the re-entering people, who have better health, from the non-employed group results in lowering health outcomes (general health, physical functioning and mental health) and increasing number of health conditions of the remaining non-employed people. This partly contributes to widening health gap between those who were working and who were not.

In brief, the exit of workers with poorer health from the employed group and the re-joining of healthier older adults to the workforce is a health selection effect that increases the health gap between workers and those who were not working. The movement into and out of the labour market by older people with differing health status has contributed to the widening health gap.

### Widening health gaps accompanied by widening economic outcomes

The widening health gap raises the question of what it means for economic outcomes in the short term and in the long term, and for socio-economic inequality. We hypothesized that the widening health gap is accompanied by widening income, superannuation, and net financial asset gaps. In this subsection, we considered these economic outcomes: incomes without government support, incomes with government support, superannuation, and household net financial assets^3^.

#### Short-term economic outcome: income

**Fig. 5:**
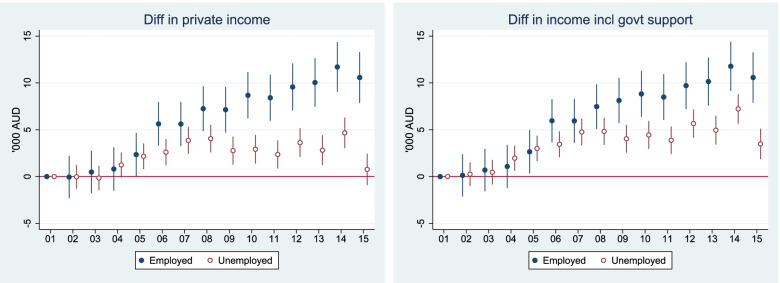
Relative change of income 2001–2015, reference year 2001, Australians aged 50 to 70. Notes: 2001 is set as the base year. All monetary variables are measured in AU$1000 and discounted to 2002 price level. Estimates were adjusted for age, sex, ethnicity, and education in the maximum likelihood RE model

Income is measured for a financial year. As shown in Fig. 5, the widening health gap (as seen in Fig. 2) is accompanied by widening income gap. There is strong evidence of widening income gap between those who were working and who were not over the considered period. When considering income with government support provided to the non-employed, the gap becomes smaller, but is still significantly large (the right panel, Fig. 5).

#### Long-term economic outcomes: superannuation and net financial assets

Over time, savings from employment-generated income helps build up superannuation and net financial assets for older workers. Prolonged employment and higher incomes contribute to larger cumulative savings, superannuation and financial assets [25, 26]. To examine the association between the economic outcomes and health in the wage labour market context, we focussed on wealth which is mainly generated from saving of labour market earnings. Investment profits (business and property investment and related debts) are not related to wage labour market activities, and have been excluded. Our measure of net financial assets captures superannuation, savings on bank accounts, bank account balances, cash and equity investments, trust funds and life insurance and reflects long term accumulated economic outcomes from wage labour market participation.

**Fig. 6:**
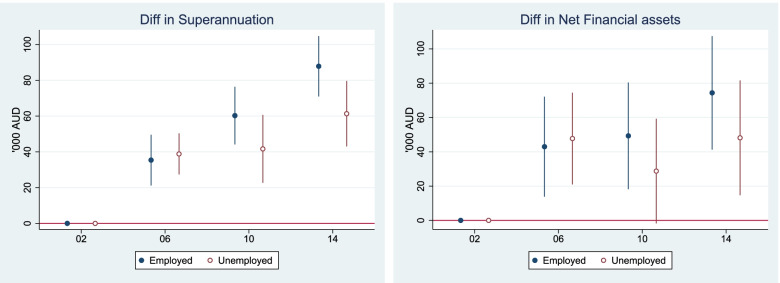
Relative change of superannuation and household net financial assets, 2002–2014 (base year 2002) Australians aged 50–70. Notes: 2002 is set as the base year. All monetary variables are measured in AU$1000 and discounted to 2002 price level. Estimates were adjusted for age, sex, ethnicity, education in the maximum likelihood RE model

Figure 6 shows how superannuation and household net financial assets change over the period 2002 to 2014 by employment status. It shows that employment boosted superannuation and net financial assets, generating a widening gap in both superannuation and net financial assets between those who were working and who were not. However, we observed large standard deviations in the changes of superannuation and the financial assets^4^; indicative that economic outcomes are heterogeneous within each group as we show in detail in the following section.

### Widening health gaps are accompanied by increasing economic inequality

#### Within and between group comparisons

The heterogeneous superannuation and net financial assets indicates that each employment status group is composed of diverse individuals. For example, in the non-employed group, some were healthy and wealthy but chose to retire early (not working). In contrast, some were not employed because they were unhealthy. The sample stratification by net wealth distribution provides insight into the heterogeneity of the sample.

Within each employment status group, we stratified the samples into the top 20% richest and the remaining 80% (also called the bottom 80% in this paper) based on net wealth distribution. We then estimated superannuation, net financial assets, and some key health measures (see Table 3 below). Among the non-employed group, the top 20% of the wealth distribution had very high net financial assets and superannuation relative to the remaining (80%) group. Their net financial assets and superannuation were close to those of the top 20% wealthiest of the employed people. Their superannuation was the highest ($578,000) among the four subgroups. This group was obviously healthy and wealthy, but chose to retire early because they had sufficient superannuation and financial assets to maintain their living standard. This group also had much better health outcomes than the rest of the non-employed group. In contrast, the remaining 80% of the non-employed group had considerably lower net financial assets, low superannuation, and poor health outcomes.

**Table 3:** Health, superannuation, and financial assets by working status, Australians aged 50–70, 2001–2015

	Employed		Not employed	
	Top 20%	Remaining 80%	Top 20%	Remaining 80%
Net financial assets	1011	206	1012	106
(AU$1000)	[958–1065]	[202–211]	[953–1070]	[102–110]
Superannuation	572	155	578	67
(AU$1000)	[544–600]	[151–158]	[543–613]	[64–69]
Physical functioning	86.9	83.5	80.0	65.0
	[86.1–87.7]	[83.1–84.0]	[78.9–81.1]	[64.3–65.8]
Mental health	80.6	77.1	78.9	70.0
	[80.0–81.2]	[76.7–77.5]	[78.1–79.8]	[69.5–70.5]
General health	73.0	69.1	66.8	54.5
	[72.1–73.8]	[68.6–69.5]	[65.7–68.0]	[53.8–55.2]
Number of health conditions	0.37	0.50	0.85	1.93
	[0.3–0.4]	[0.5–0.5]	[0.7–0.9]	[1.9–2.0]

The estimates were adjusted for sample weight, but were not adjusted for age, sex, ethnicity, and education, and 95% confidence intervals are in brackets

The top 20% of those who were not employed were ‘wealthy and healthy’. This subgroup had about 6 times more net wealth as that of the remaining 80% of the non-employed, 10 times the net financial assets, and 9 times the superannuation of the remaining 80% of the non-employed. For this subgroup, irrespective of their health, their wealth protected them financially. However the remaining 80% of the non-employed were both ‘un-wealthy and unhealthy’.

The top 20% of employed older Australians were similar to the top 20% of the non-employed in terms of health, net financial assets and superannuation. The key difference is that they chose to remain in the labour market. In contrast, the remaining 80% of the employed were less wealthy but they were relatively healthy. Their health outcomes were close to those of the top 20% of both the employed and non-employed groups. Their better health hence enabled them to remain in the labour market.

That the estimates in Table 3 have large standard deviation (we do not report here instead of confidence interval for easier comparing across groups, but will be available upon request) is expected for variables such as superannuation, because it reflects the large variance within a narrow subgroup (using wealth distribution) and skewness of the data (skewed to the right). Within the richest (top 20% of wealth distribution), we still see some with very low superannuation while the others have a very high superannuation balance. This suggests that sources of wealth are heterogeneous. For example, for some people their main source of wealth is their employment income (these people have high superannuation) while for others the main source of wealth is from non-wage employment such as investment income, properties etc. (these people have low superannuation).

**Fig. 7:**
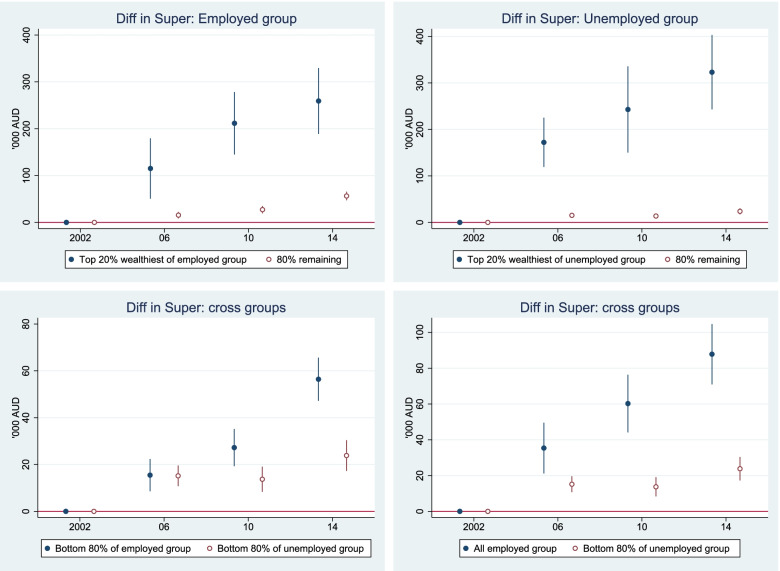
Relative change in superannuation 2002 to 2014, reference year 2002, Australians aged 50 to 70. Notes: Estimates were adjusted for age, sex, ethnicity, and education in the maximum likelihood RE model. The reference year is the first year that the data are available. Superannuation was first collected in 2002, and measured in AU$1000 and discounted to 2002 price level

Figure 7 considered how superannuation changed over the period using 2002 as the baseline. The top left panel compares the top 20% wealthiest and the remaining 80% of the employed group. It shows a widening gap in superannuation, though the remaining 80% subgroup’s superannuation has improved over time. The gap in superannuation between the top 20% and bottom 80% of the non-employed group has grown faster than the gap between the top and bottom of the employed group. This again confirms that the wealthy and healthy within the non-employed chose to retire early or chose not to work in the wage labour market because they had sufficient superannuation and other sources of income.

The bottom right panel compares superannuation changes between all employed workers and the remaining 80% of the non-employed group (the un-wealthy and unhealthy). The bottom left panel compares superannuation changes over time between the “unhealthy and un-wealthy” in the non-employed group and the “healthy and un-wealthy” in the employed group. This comparison best demonstrates the role of health in employment and cumulative saving/superannuation. It suggests that better health promotes employment participation among older people which in turn helps build up superannuation. The gap in superannuation between these two subgroups has widened over the period.

**Fig. 8:**
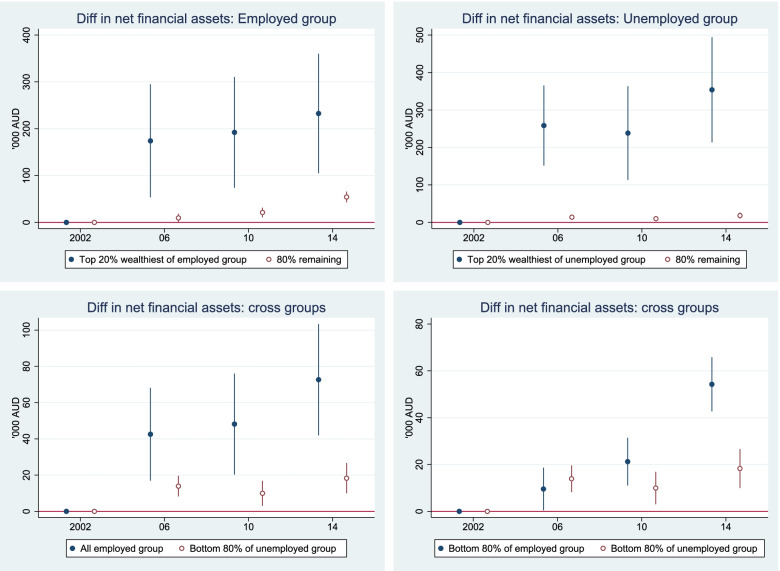
Relative change in net financial assets from 2002 (base year) to 2014, Australians aged 50 to 70. Notes: Estimates were adjusted for age, sex, ethnicity, and education in the maximum likelihood RE model. The reference year is 2002. Net financial assets were computed from bank account balance, superannuation, cash and equity investment, trust fund, life insurance, credit card debts, and other related debts. As some of these measures were first collected in 2002, and only collected in 2002, 2006, 2010 and 2014, we thus set 2002 as the base year and the Figure only shows data in those years. The measure is in AU$1000 and discounted to 2002 price level

In Fig. 8, we repeated the same analysis for net financial assets as for superannuation in Fig. 7 and found a similar pattern. The gap in net financial assets has widened over time within each working status group (top two panels of Fig. 8), and across groups (bottom two panels). Remaining employed due to better health helps build up financial assets faster than not being employed (bottom left panel). The unhealthy and un-wealthy non-employed group face the double hazards of poor health, and low or little improvement in superannuation and net financial assets.

#### The un-wealthy and unhealthy group left behind – worsening health and economic inequality

The *‘left-behind’* group (the un-wealthy and unhealthy), a remaining 80% of the non-employed, is facing a growing health and economic gap. They endure poor health and have no opportunity to improve their income. Below we have provided further evidence that the un-wealthy and unhealthy group has declining resources to improve their health and wellbeing.

### Within the non-employed group comparison

**Fig. 9:**
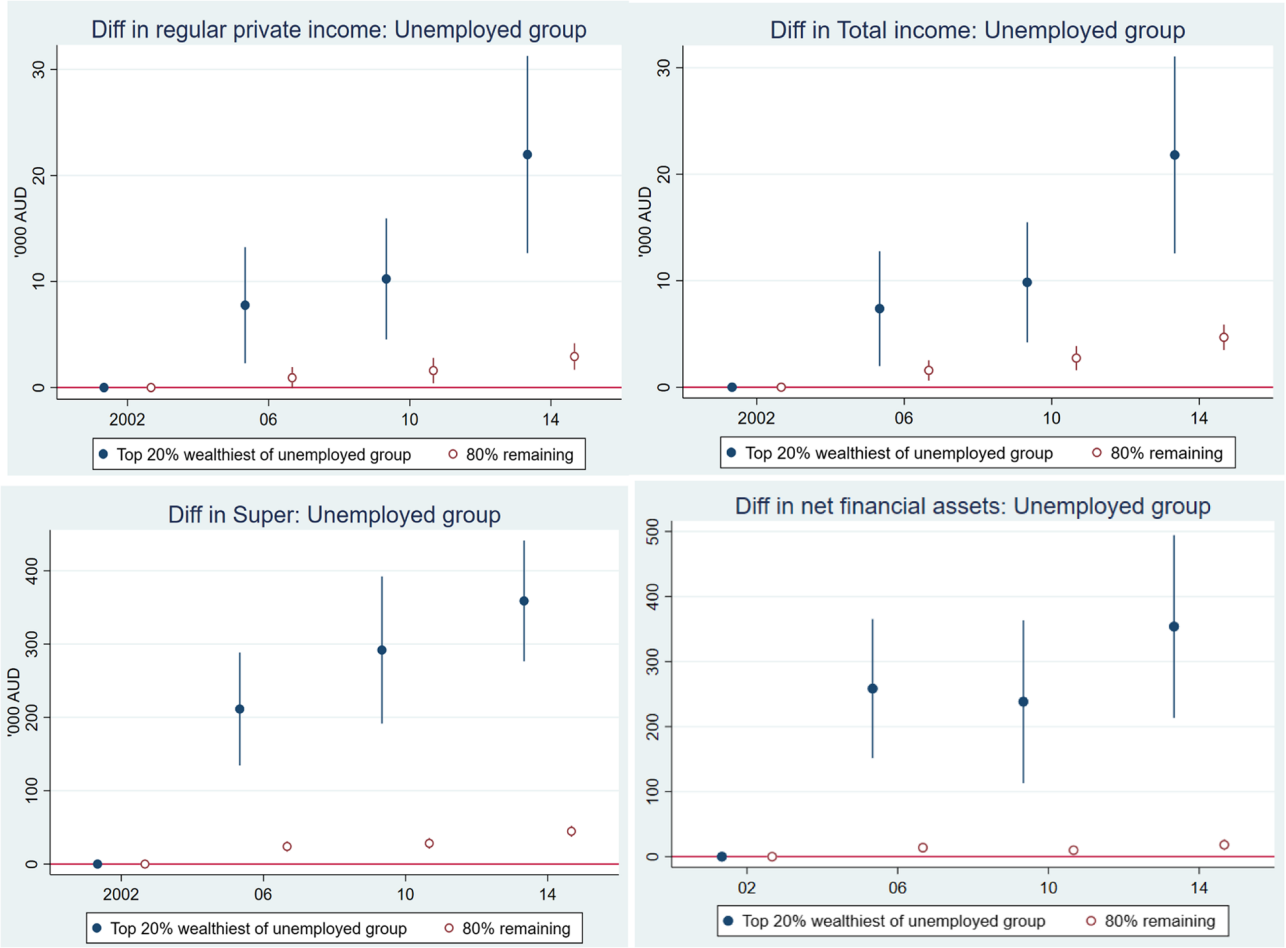
Rising economic inequality within the non-employed group 2002–2014 (base year 2002) Australians aged 50–70. Notes: Estimates were adjusted for age, sex, ethnicity, and education in the maximum likelihood RE model. The reference year is the first year that the data are available. Superannuation was first collected in 2002, and measured in AU$1000 and discounted to 2002 price level

In Fig. 9, we compared four economic outcome changes between the top 20% wealthiest and the remaining 80% (the left-behind) of the non-employed. The economic outcomes are regular private income, total income (regular private income and government supports such as pension and social welfare benefit), accumulated superannuation, and household net financial assets.

**Fig. 10:**
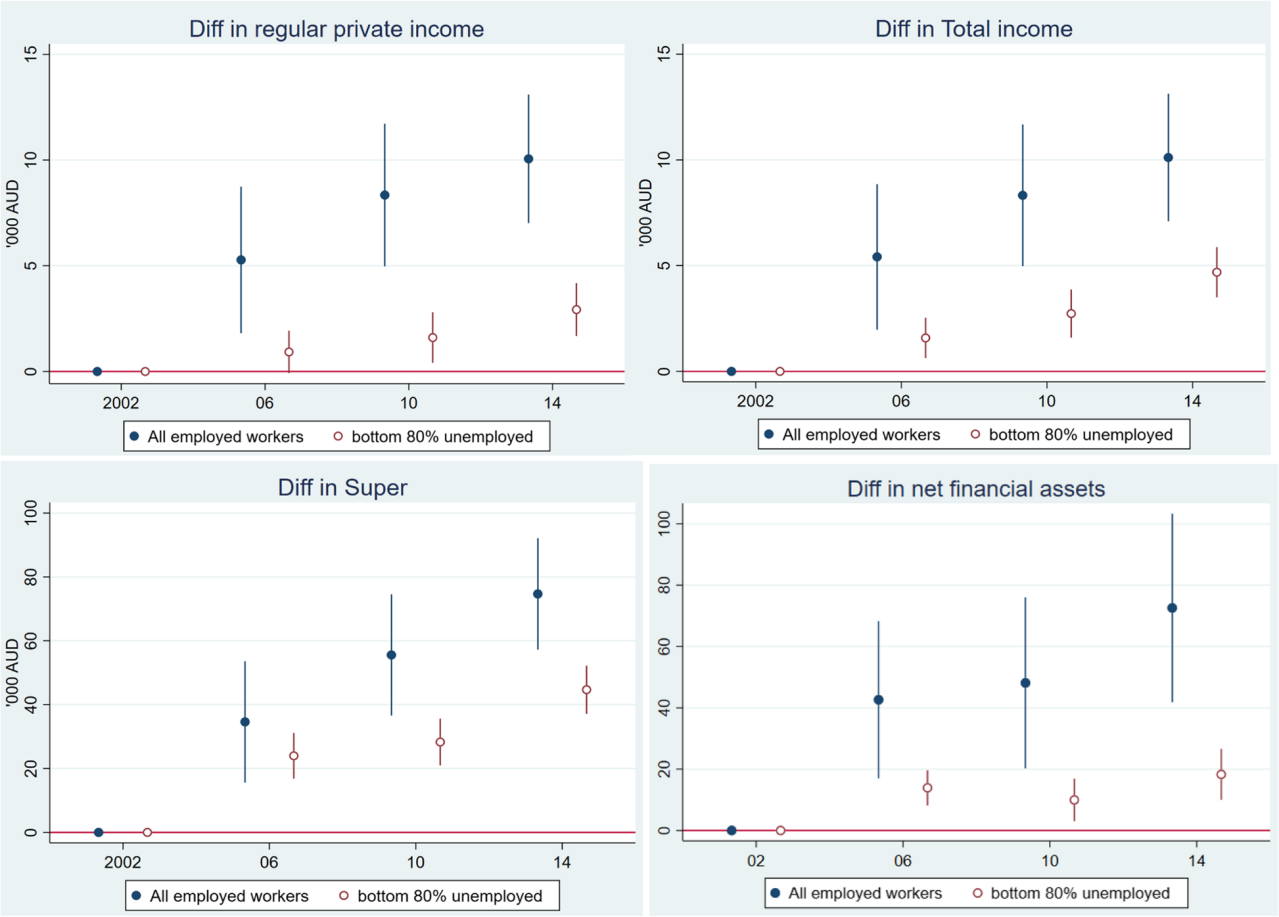
Rising economic inequality between the ‘*un-wealthy and unhealthy’* and “*employed group”* 2002 to 2014 (base year 2002), Australians aged 50 to 70. Notes: Estimates were adjusted for age, sex, ethnicity, and education in the maximum likelihood RE model. Superannuation was first collected in 2002, and measured in AU$1000 and discounted to 2002 price level

Compared with the baseline year 2002, the wealthiest of the non-employed group received more than $20 k income in 2014, while the ‘left behind’ group’s income changed very modestly. The addition of the government support made a small change to the total income over the period, of just less than $5 k. In terms of superannuation and net financial assets, the left behind group own relatively small amounts of superannuation and financial assets in relation to the wealthiest, and the gap between these two groups has become very large (bottom panel of Fig. 9). Figure 10 compared the changes in economic outcome over time for the un-wealthy and unhealthy group with all employed older workers. It again shows widening gaps in income, superannuation and net financial assets. The employed group’s income, superannuation and net financial assets improved over time at a greater rate than those of the left-behind group.

Briefly, considering all the comparisons we have made so far, the common finding is that those who had poor health and were un-wealthy are increasingly falling behind in all economic outcome measures such as income, income with the government support, superannuation and net financial assets. They did not, and likely could not, enter the labour market to mitigate disadvantage as they aged.

### Consolidated analysis

The current analysis does not aim to estimate causal relationships but instead examines the association between health and employment participation. Linking health differentials by work status may face endogeneity biases due to reverse causality. To address this and strengthen our policy discussion we analysed older workers’ labour market participation using their *prior* health measures (1st lags) as predictors.

Using a longitudinal logistic model, all prior health measures strongly affected the probability of labour market participation for 50–70 year olds, supporting the key role health status plays for selection into the labour market (Table 4). The estimates show that prior better health measures are good predictors of being employed for older workers. More specifically, the negative coefficients e.g., having fair or poor health (y = 1) or having a health restriction (y = 1) will reduce the likelihood of being employed, while the positive coefficients e.g. mental health, general health or physical functioning means better health (in these health measures) and will increase the likelihood of being employed.

**Table 4:** Health conditions and working status, Longitudinal (RE) Logistic model, Australians aged 50–70, 2001–2015

	(1)	(2)	(3)	(4)	(5)	(6)
Fair & poor health (lag) (yes = 1)	−1.1437***	− 0.9717***	− 0.5541***			
	(0.0832)	(0.0854)	(0.1000)			
Mental health (lag)		0.0199***	0.0141***	0.0141***	0.0162***	0.0170***
		(0.0022)	(0.0023)	(0.0023)	(0.0022)	(0.0022)
Gen health (lag)			0.0197***	0.0263***		
			(0.0025)	(0.0021)		
Health restriction (lag)					−1.5091***	
(Yes = 1)					(0.0791)	
Physical functioning					0.0188***	0.0212***
(lag)					(0.0018)	(0.0018)
Long term health						−0.9517***
Condition (lag) (yes = 1)						(0.0682)
Constant	31.52***	30.25***	28.72***	28.28***	27.57***	27.47***
	(0.7043)	(0.7113)	(0.7218)	(0.7102)	(0.7182)	(0.7189)
Prob>chi2	0.0000	0.0000	0.0000	0.0000	0.0000	0.0000
Observations	38,512	38,360	37,992	38,401	38,421	38,434
No of individuals	6928	6923	6910	6915	6920	6920

Standard errors in parentheses, *** *p* < 0.01. Models also controlled for lags of net financial assets, financial distress, non-wage income, age, unpaid time, marital status, gender, education level, ethnicity, state, and year dummy variables

Moreover, an assessment that health did not improve (after adjusting for many factors, including equivalised income per capita) for workers and non-workers may be problematic due to the risk of endogeneity from 2-way causality between income and health. When we trialled models using *prior* equivalised income per capita, the estimates did not change the health trend over time (the estimates are available upon request).

## Discussion, limitation and conclusion

This paper has examined the interplay between the older population’s health, wealth and employment participation over the past fifteen years. It showed that Australia’s participation policy has been successful; the older labour force increased by 10 % during the past 15 years. However, we found little evidence of improvement in health outcomes for this older population regardless of their working status. In most instances, we observed health deterioration over the period. Overall, healthier older Australians remained in employment or returned to the labour market if they were previously non-employed, while those with poorer health remained non-employed or exited the labour market if they were employed. The health gap between those working and those who were not has widened over the short 15 year period of this study. The increasing rates of people with relatively better health returning to the labour market, and increasing rates of people with relatively poorer health exiting the labour market, operates as a dynamic process of health selection which has contributed to the widening health gap between the employed and the non-employed people.

The widening health gap between those who were working and those who were not is accompanied by rising economic inequality within the older population. Income, net financial assets, superannuation, and health interact. Better health enables people to remain or return to the labour market, while poor health keeps or pushes them out of the labour market [27, 28]. The financial benefits of employment assist older workers maintain their good health or impedes health deterioration due to their ability to pay for healthcare and other health-supportive resources [29]. Net financial assets and superannuation protects the older people’s health as they age further. Older people who were healthy and wealthy, arguably the most advantaged in the population, can therefore choose to remain employed or retire early because they have sufficient accumulated assets and superannuation. In contrast, older people with poor health (which has likely already limited assets and superannuation), require income and employment for financial reasons, but their poor health prevents them from labour market participation.

The current aging and workforce participation policy in Australia does not address the health needs of many older adults. The pension age eligibility of 67 in Australia is realistic for some, but as we show problematic for many other older workers, particularly for those who have poorer health. While increasing longevity enables people to work longer in principle, but its success, and social equity, pivots on health status.

Our analysis considers the inter-relationships between older workers’ health and workforce participation rates over the past 15 years. However, our analysis does not examine the causal relationship between health and employment participation because we did not address the reverse relationship whereby employment participation may improve health outcomes. The main limitation of this study is that we demonstrate an association between health and employment but cannot show causality. However, we consolidate this finding through additional analysis (see Table 4) that strengthens a plausible causal inference.

In this paper, we have provided new evidence to support a potential policy debate. First, we showed, in the context of increasing longevity, that overall health is either or improving or deteriorating, including among those who remain in the labour market. Policies that promote jobs and businesses to accommodate the diverse health needs and capabilities of their older workforce are required, as are polices that focus solely on protecting and promoting health to enable all to work as well as live longer. Second, rising health inequality between the employed and non-employed is accompanied by rising economic inequality in incomes, cumulative financial assets and superannuation. As we showed in this analysis, improving the older population’s health is one of the keys to promoting employment participation as well as reducing the need for financial support and thus mitigating public financial pressure. Third, although some older non-employed adults were healthy, they can choose not to work due to their high wealth, assets and superannuation, while the majority of non-employed generally had low incomes, low assets and low superannuation and poor health. This group had both greater need for financial support and security and far less capacity to gain it from employment. Policy may need to rethink how and what types of employment participation are feasible, and what else is needed to support those who are unable to work and to prevent this group growing in the future.

## Supplementary Information

**Additional file 1.** Household net wealth (including property) 2002–2014 for Australians aged 50–70 by working status.

## Data Availability

We are not allowed to share the unit-record data, however, the dataset is accessible to anyone with an approval from HILDA.

## Abbreviations

HILDA: Household Income and Labour Dynamics in Australia Survey
DSS: Australian Government Department of Social Services
CPI: Consumer Price Index
OECD: The Organization for Economic Co-operation and Development
FE: Fixed Effect model
SD: Standard Deviation

## Footnotes

1 Adjusting household incomes: equivalence scales (oecd.org)

2 We standardized (by mean and standard deviation) all health variables in order to put the health outcomes in the same range figures for convenient comparison. The estimated coefficient when using standardized dependent variable Y is called *standardized* regression coefficient, and it represents the expected change in *Y* (in standardized units of *S*_*Y*_ where each “unit” is a statistical unit equal to one standard deviation) due to an increase in *X*_*i*_ of one of its standardized units (*S*_*Xi*_), with all other X variables unchanged. Each standardized regression coefficient is in units of standard deviations of *Y* per standard deviation of *Xi*.

3 Note that we do not add up superannuation or net financial assets with the income as income is measured for a financial year, while superannuation or net financial assets are stock and accumulated over time.

4 Property or housing assets may be also important when considering the economic outcome gap between working and non-working mature aged people. Property and business assets are strongly influenced by non-labor market factors such as household business performance and the housing market. Particularly in Australia, household wealth is strongly affected by house ownership due to the current hot housing market. Furthermore, when property-related debt increases fast (even faster than property value) it leads to a decline in household net property assets which ultimately affects household net wealth (see Additional file 1). Using net assets or net wealth including business and property assets (non-financial assets) does not capture well health-labour market outcome relationship (note that net assets include net financial assets and net non-financial assets). For these reasons, we do not consider an economic measure that includes net property and business assets.
