# Supplementary material for: Workforce participation, health and wealth inequality among older Australians between 2001 and 2015
Source: Arch Public Health. 2022 Mar 31;80:104. doi: 10.1186/s13690-022-00852-z (PMC8969371; doi:10.1186/s13690-022-00852-z)
Supplement: Supplementary file 1 — Additional file 1. Household net wealth (including property) 2002–2014 for Australians aged 50–70 by working status. [file 13690_2022_852_MOESM1_ESM.docx]

Additional file 1: Household net wealth (including property) 2002-2014 for Australians aged 50-70 by working status.

*Note: all assets and its related-debt variables were discounted to 2002 price level. Note that we used real terms so one may see housing price increased year on year elsewhere, but here we adjusted for inflation the real housing price growth is thus low from mid 2000s to 2014.*
